# COUPLES’ FINANCIAL BEHAVIORS AND SATISFACTION AMONG KOREAN BABY BOOMERS

**DOI:** 10.1093/geroni/igac059.2332

**Published:** 2022-12-20

**Authors:** Youngwon Nam, Kyungmin Kim, Gyounghae Han

**Affiliations:** Seoul National University, Seoul, Seoul-t'ukpyolsi, Republic of Korea; Seoul National University, Seoul, Seoul-t'ukpyolsi, Republic of Korea; Seoul National University, Seoul, Seoul-t'ukpyolsi, Republic of Korea

## Abstract

The life-cycle theory posits that midlife is a peak time of earning and productivity. However, middle-aged adults also face complex financial decisions (e.g., mortgage, child tuition cost, care expenses for older parents, and their own health care expenses), which may influence their current and future financial well-being. While research has focused on individuals’ financial management behaviors at midlife, less is known about the interdependence of financial behaviors and well-being within a couple, which may be a more relevant unit of financial decisions. To address this gap, we examined middle-aged couples’ financial behaviors and satisfaction, using a sample of 1,111 couples (age 51–59) from the 2014 Korean Baby Boomer Panel Study (KBBPS). Wives showed consistently higher levels of financial behaviors in (a) recognizing financial status, (b) planning and monitoring financial goals, and (c) building and maintaining household wealth. Korean Baby Boomer couples also showed substantial within-couple similarity in planning and monitoring financial goals (ICC = .51) and building and maintaining household wealth (ICC = .65)—whereas they showed less shared awareness of household financial status (ICC = .23) between spouses. Results from the Actor-Partner Interdependence Models (APIM) revealed that beyond one’s own financial behaviors, their spouses’ financial behaviors were associated with higher financial satisfaction for both husbands and wives (partner effect). Further, intra-couple differences in financial behaviors were associated with lower financial satisfaction only for wives. These findings highlight the importance of shared process of financial decision making for financial well-being among couples in middle and later life.

